# Nanoscale imaging of untreated mammalian cells in a medium with low radiation damage using scanning electron-assisted dielectric microscopy

**DOI:** 10.1038/srep29169

**Published:** 2016-07-04

**Authors:** Tomoko Okada, Toshihiko Ogura

**Affiliations:** 1 Biomedical Research Institute, National Institute of Advanced Industrial Science and Technology (AIST), Central 2, Umezono, Tsukuba, Ibaraki 305-8568, Japan

## Abstract

Imaging of untreated living cells in a medium at a nanometre-scale resolution under physiological conditions is a significant challenge. Scanning electron microscopy (SEM) is widely used to observe cells in various atmospheric holders or special equipment. However, untreated biological specimens in aqueous solution generally incur heavy radiation damage from the direct electron beam (EB); and these images exhibit very poor contrast. Therefore, a new method for generating high-contrast images of living cells under physiological conditions without radiation damage has been strongly desired. Here, we demonstrate the first nanoscale observation of living cultured mammalian cells using our newly developed scanning-electron assisted dielectric microscopy (SE-ADM) method with a culture dish holder. Using the difference in relative permittivity between water and specimens, our SE-ADM system aids in the visualisation of untreated biological samples in aqueous solution. In addition, specimens incurred only a low level of radiation damage because the tungsten (W)-coated silicon nitride (SiN) film absorbs irradiated electrons. Untreated cells and organelles are clearly visible in high-contrast and high-resolution images without staining and fixation. Furthermore, our method enables the detection of changes in organelle structures within cells via time-lapse imaging with minimal radiation damage.

Nanometre-scale visualisation of living cells can provide valuable insights into biological mechanisms^1^,^2^,^3^. Recent fluorescence microscopy techniques involving super-resolution technology with fluorescent labelling of cellular components enable resolutions of approximately 20 nm^4^,^5^,^6^, but these techniques only enable observation of labelled cellular regions. In contrast, conventional SEM yields spatial resolutions >10 nm. However, SEM requires fixed and/or frozen or dried cells and does not reveal information under physiological conditions^7^,^8^,^9^. The EB used in electron microscopy studies of eukaryotic cells in medium, which involved atmospheric holders, caused heavy radiation damage to the cells, resulting in a requirement of glutaraldehyde fixation with negative staining or metal labelling^10^,^11^.

X-ray free-electron laser technology was recently established^12^,^13^,^14^. Using this technique, unstained and unfixed living biological samples can be visualised using diffraction patterns and electron densities reconstructed with femtosecond X-ray laser pulses^15^,^16^. Although this method has been successfully used to observe viruses and living prokaryotic cells^12^,^13^,^14^, the biological samples were completely destroyed after a single-shot X-ray pulse^16^. In addition, X-ray laser systems require huge equipment and involve high costs^13^,^14^. Therefore, a new method for observing living cells under physiological conditions without radiation damage has been strongly desired.

Recently, we developed a new imaging technology involving a SE-ADM system, which comprises a SEM, electric-field detection system, and aqueous sample holder^17^,^18^,^19^. Using this system, we can visualise untreated biological samples in aqueous medium using the differences in relative permittivity (RP) between the substances. The RP of water is approximately 80^20^ or 30-fold higher than that of biological specimens (RP = 2–3)^21^. Therefore, our system is capable producing high-contrast images of untreated biological specimens in aqueous solution^17^. In this process, biological samples are enclosed in a liquid holder composed of W-coated SiN film and are not directly exposed to EB, thus minimizing electron radiation damage^17^. When the electron beam is used to irradiate the 15-nm W layer, the electrons are scattered and absorbed in this layer; hence, a negative potential arises in the irradiated position. This negative potential is detected from the bottom measurement terminal through the specimen in water^17^. Our system, which is based on high-resolution field emission (FE) SEM, has a spatial resolution of 8 nm^19^. Moreover, our method enables the detection of changes in organelle structures within cells via time-lapse imaging with minimal radiation damage.

## Results

**Figure 1:**
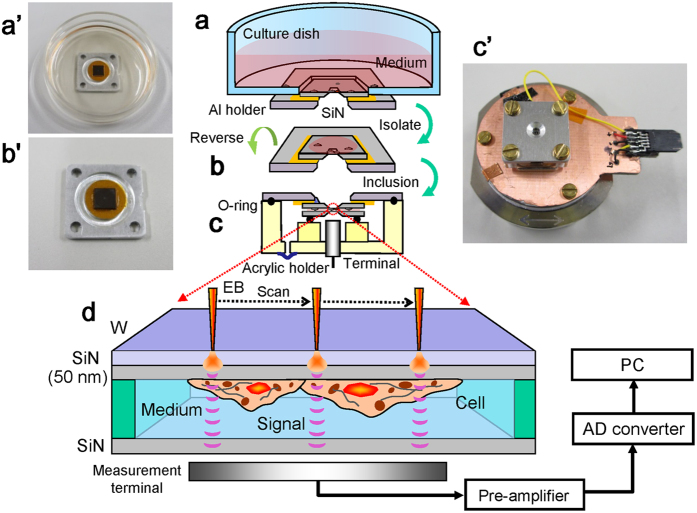
Experimental set-up and dielectric microscopy using a culture dish holder and SE-ADM system. (**a**,**a’**) Al holder with W-coated SiN film is attached in the bottom of a medium-filled culture dish. After 4–5 days of culture, cells in the holder formed a confluent monolayer on the 50-nm-thick SiN film. (**b**,**b’**) The holder containing cultured cells was separated from the plastic culture dish after removing part of the medium. (**c**,**c’**) The Al holder was attached upside down onto another SiN film on a square acrylic plate. These components were sealed with an O-ring and four screws. The sample holder with cells in the medium was mounted on the pre-amplifier attached to the sample stage. (**d**) Schematic figure of our high-contrast imaging method with low-level radiation damage to untreated cells in the medium under W-coated SiN film. EB irradiation of the W-coated SiN film causes electron scattering and absorption in the film, and negative potential in EB-irradiated position. This negative electric potential is transmitted to the bottom SiN film through the cultured cells in the medium.

Figure 1 presents a schematic outline of the SE-ADM system and the original culture dish with liquid sample holder (Fig. 1a,a’). The holder containing cultured cells was separated from the plastic culture dish (Fig. 1b,b’) and attached upside down onto another SiN film on a square acrylic plate equivalent in size to the Al holder (Fig. 1c,c’). These components were sealed with an O-ring and four screws (Fig. 1c). Cultured mammalian cells in the interspace between the SiN films were maintained at atmospheric pressure (Fig. 1d). The liquid sample holder, including the intact cultured cancer cells (4T1E/M3), was attached to a sample stage with a built-in pre-amplifier (Fig. 1c’). This assembly was placed in a FE-SEM chamber via a sample transfer system. W-coated SiN film on the cell-adherent side was irradiated by a scanning-EB at an acceleration voltage of 7–10 kV. A measurement terminal under the sample holder detected electrical field signals transmitted from EB-irradiated position on the W-coated SiN film to the cells in the medium (Fig. 1d). Electrical field signals are strongly transmitted through the medium to the underside. In contrast, cells strongly inhibit these signals. Finally, dielectric images were generated via calculations involving the electrical-field and EB-scanning signals^19^.

We analysed the electron trajectories on W-coated SiN film and in the medium using the Monte Carlo (MC) simulation in CASINO ver. 2.42^22^. We estimated that the density and thickness of the W layer and SiN film were 19.3 g/cm^3^ and 15 nm and 3.12 g/cm^3^ and 50 nm, respectively. The calculated density of the culture medium was 1.012 g/cm^3^, because RPMI-1640 medium mainly included NaCl (6 g/L), NaHCO_3_ (1.5 g/L) and D-Glucose (4.5 g/L). The simulation parameters were set as follows: 100,000 electrons, 8 kV acceleration voltage, and 3-nm EB diameter. As a result, the trajectory of the irradiated electrons was found to reach a depth of approximately 1 μm in medium (Supplementary Fig. 1a,b), whereas the mean thickness of the 4T1E/M3 cells is estimated at approximately 3 μm^23^. Next, we calculated the total electron doses to the cells in medium based on the EM condition and MC simulation. The electron dose just below the SiN membrane was 0.335 electron/Å^2^, which was calculated using a MC simulation with EB current of 10 pA, EB scanning area of 12 × 9.6 μm and transmission rate of 0.774 (Supplementary Fig. 1c,d). The electron dose to the cell gradually decreased according to depth from the SiN film (Supplementary Fig. 1d), reaching a value near 0 at a depth of 1.2 μm, in comparison to the cell thickness of approximately 3 μm^23^. Therefore, the cell appeared to sustain minimal radiation damage.

**Figure 2:**
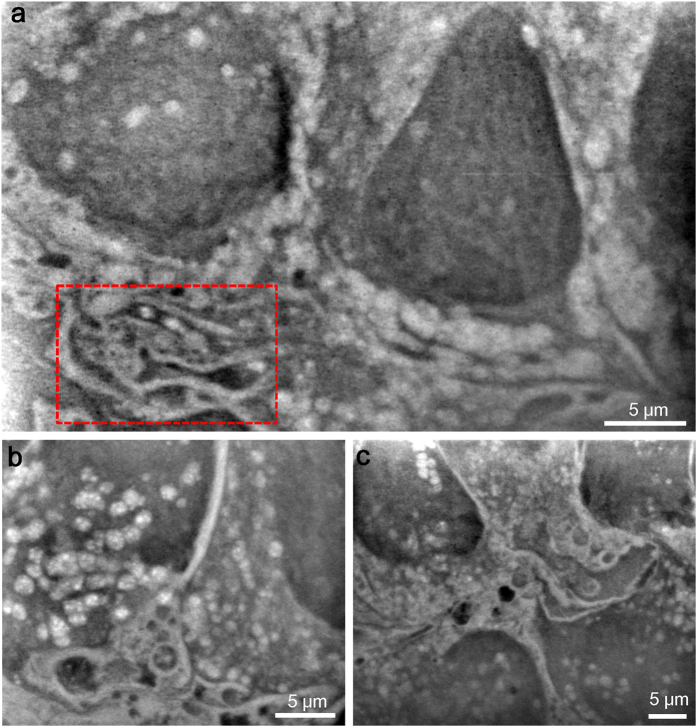
Observation of untreated 4T1E/EM3 cells in the medium using SE-ADM system. (**a**) Dielectric image of untreated culture cells in medium at 2,500× magnification, with a 7-kV EB and −32-V bias. ERs are visible as a complex membrane stack in the lower left (boxed region). (**b**) Dielectric image near the nuclear regions. The membrane and vesicle structure are shown at the lower left. Many vesicles are dispersed throughout the regions. The image was obtained with a 9-kV EB, 4,000× magnification and −32-V bias. (**c**) Dielectric image of other membrane and vesicle structure regions obtained at 8 kV and 2,500× magnification. All scale bars represent 5 μm.

After 4–5 days in culture, cells in the holder formed a confluent monolayer on SiN film and were observed using an optical phase-contrast microscope (Supplementary Fig. 2a). We seeded the cells into the holder dish at a rather low density and allowed them to adhere and spread because we can obtain clearer dielectric images with our SE-ADM system with a thinner sample. We initially observed the SiN film under the cells using a dielectric microscope at low magnification. The upper sides of W-coated SiN films were visualised using secondary electron images, which detected flat film surfaces without cells (Supplementary Fig. 2b). In contrast, dielectric images revealed several cancer cells in the SiN film square window (Supplementary Fig. 2c). Furthermore, our system clearly visualised cancer cells at 2,000× magnification (Supplementary Fig. 2d). Another representative image at 2,500× magnification (Fig. 2a), intracellular structures, including the nuclei, vesicles, and endoplasmic reticulum (ER), were clearly observed. Typically, the nucleus appeared as a large spherical structure with strong black contrast. Our SE-ADM system is able to observe specimens at a medium depth of approximately 10 μm^17^.

The ER, which is located within the cytoplasm, is a membrane network composed of branching tubules and flattened disc-like sacs^24^,^25^. ERs were found to localise near the nucleus in 4T1E/M3 cells stained with fluorescence dyes specific for the ER and Golgi complex and observed using optical fluorescent microscopy (Supplementary Fig. 3a). The phase contrast image and its merged picture are shown in Supplementary Fig. 3b,c. We used SE-ADM imaging to view the intracellular structures of intact 4T1E/M3 cells in medium. ER under the nucleus exhibits complex membrane structures (Fig. 2a, boxed region). Figure 2b shows a region near the nucleus at 4,000× magnification, with the nucleus appearing as a spherical area of dark contrast, surrounded by tubule-like structure of the ER and large vesicles. A similar complex membrane structure was observed in another area (Fig. 2c).

**Figure 3:**
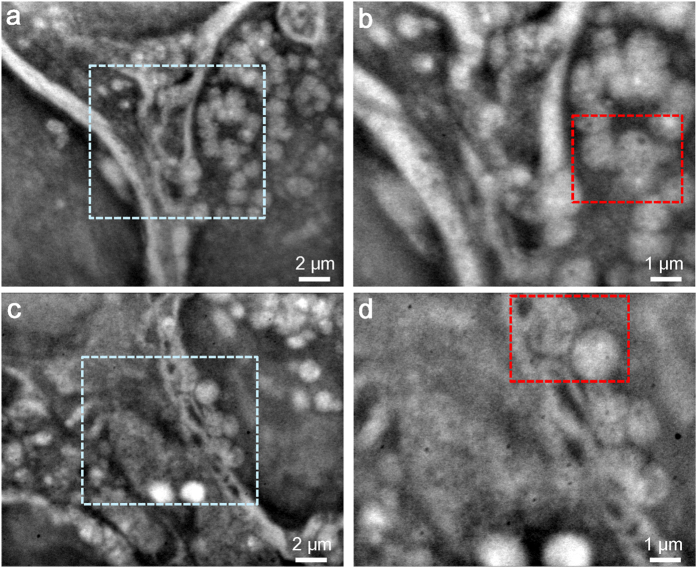
High-resolution imaging of vesicle structures using SE-ADM. (**a**) Dielectric image of a vesicle-rich region. Clear vesicles are visibly attached to membrane structures. The image was obtained with a 9-kV EB, 5,000× magnification, and −32-V bias. (**b**) High-magnification image of the boxed area in (**a**) at 10,000× magnification. Vesicles are visibly attached to the membranes. (**c**) Dielectric image of another vesicle-rich region at 5,000× magnification. (**d**) High-magnification image of the centre of (**c**) at 10,000× magnification. The vesicles are clearly attached to membrane -like structures. The scale bars represent 2 μm in (**a**,**c**) and 1 μm in (**b**,**d**).

The ER plays important roles in the biosynthesis of proteins and lipids; these molecules are transported from the ER to the Golgi complex which process has been precisely studied^26^. The structures of the ER and Golgi complex have been observed using high-resolution TEM^27^. SE-ADM images of 4T1E/M3 cells (Fig. 3) indicate various vesicles and/or ER (white contrast) near the nucleus and membrane structures (Fig. 3a,c, 5,000×). High-magnification (10,000×) scans of central areas revealed vesicles attached to membrane-like structures (Fig. 3b,d). Two enlarged vesicle and/or ER images of red-boxed areas from (b,d) (Supplementary Fig. 4a,b) clearly reveal a spherical shape with rough surface membrane. Furthermore, the vesicles were found to be interconnected.

**Figure 4:**
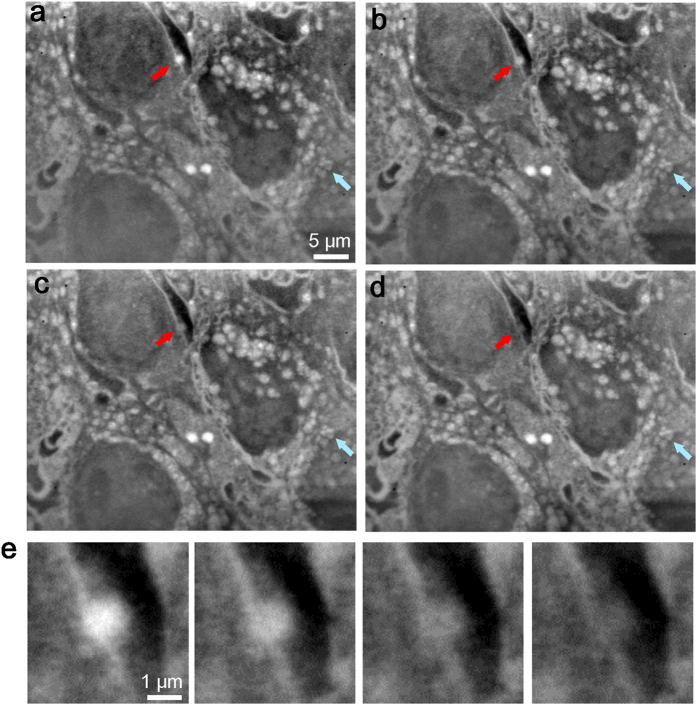
Examination of structural changes within cells using multi-scanned SE-ADM images. (**a**) Initial image of untreated cultured cells in medium using an 8-kV EB and 2,500× magnification. (**b**) Second observation image of the same area as in (**a**). (**c**) Third observation image. (**d**) Fourth scanned image of the same cells in medium. Red and blue arrows correspond to moving structures and/or changes in contrast. (**e**) Enlarged images of a vesicle that exhibited contrast changes throughout the four observations, as indicated by the red arrows in the upper parts of (**a**–**d**). The scale bars represent 5 μm in (**a**) and 1 μm in (**e**).

Our dielectric microscopy system allowed observations of intact cells in the medium with minimal radiation damage, facilitating the detection of structural movement and/or contrast change through multiple scans of the same cells. We successfully scanned the same cells four times at an approximate 6-minute interval with very little radiation damage (Fig. 4a–d). Several cells were located in the area shown in the first scanned image (Fig. 4a). The four scanned images appeared quite similar (Fig. 4a–d), indicating that our imaging method causes low levels of radiation damage to untreated mammalian cells in the medium. After a precise analysis of the first and last images, we found that the same cells under the same conditions exhibited slight changes (Fig. 4a–d, red and blue arrows). These contrast shifts and structural changes were clearly confirmed in enlarged images (Fig. 4e and Supplementary Fig. 4c). A white particle (red arrow) in Fig. 4a clearly decreased across the four scanning time points (Fig. 4e). In contrast, the contrast levels of several vesicles (Fig. 4a, blue arrows) increased, and two central vesicles fused (Supplementary Fig. 4c). We assumed that the very small changes visible in time-lapse images (Fig. 4a–d) were indicative of changes in the condition of the cell in the liquid holder rather than radiation damage. Radiation damage should be visible throughout the cell, and cells that incur greater damage will degrade. However, the detected changes were highly localized.

## Discussion

During general SEM examination, biological samples in aqueous solution in a liquid sample holder exhibit very low contrast because of nearly the same EB interaction between water and biological specimens. Therefore, it is difficult to obtain high-resolution and high-contrast images of untreated cells in the medium using a traditional liquid holder (Supplementary Fig. 5). Moreover, EB used in standard methods causes significant radiation damage to biological samples^2^,^28^,^29^. In contrast, our new SE-ADM system enables the examination of untreated biological samples in aqueous solution with little radiation damage because EB does not directly irradiate the samples^17^,^18^,^19^.

Using our new SE-ADM system, we successfully observed not only intact culture cells in the medium but also intracellular organelles. Notably, our system has a spatial resolution of 8 nm^19^, which is sufficient for the detailed analysis of cell organelle structures. Using a traditional electron microscope, whole-cell observations based on transmitted images are very challenging because it is difficult to transmit electrons to the cells in a sample thickness >1 μm. In contrast, our SE-ADM system can clearly visualise the intracellular structures of untreated whole mammalian cells in the medium (Figs 2 and 3). Moreover, living cells incurred only low levels of radiation damage from EB, even after several scans (Fig. 4), indicating the ability of our system to detect changes in living cells in medium. Notably, our SE-ADM system is the first to successfully detect changes in intracellular structures (Fig. 4a–d), such as decreasing vesicle contrast (Fig. 4e) and vesicle fusion (Supplementary Fig. 4c). However, very rapid and dramatic changes of cell structures may be difficult to detect because the present system requires 80 s for each scan. Furthermore, in our system, cells incur slight damage during the processes of dish holder application and observation. Therefore, to detect the quick and precise change of the cell structure, development and improvement of new detection system is still required. Because AFM and EM have been used to observe vesicle fusion and Golgi complex budding^30^, it might be interesting to analyse these phenomena using our system together. Further, we plan to keep the temperature in the sample stage holder at 37 °C for the long-term time-lapse imaging of living cells in the future.

Using our SE-ADM system, the spatial resolution of the cells gradually decreased in deep regions from the W-coated SiN film. Presumably, the electric field signal gradually spread and decreased in these deep regions, causing decreases in spatial resolution and contrast. To avoid an expansion of the electric potential signal range, we could generate an electric field in the holder and create an electrostatic lens using electric potential, thus condensing the range in the deep area. Therefore, we plan to include several bias-electrodes in the holder, apply voltage and form a virtual electrostatic lens. Observation of macro-proteins can be possible if we could achieve a spatial resolution <5 nm in our system. This achievement would further expand the new field of true visualisation of macromolecules in living cells. Finally, our method could be applied to visualise various liquid samples in a broad range of scientific fields, including nanoparticles, organic materials and other biological specimens.

## Methods

### 4T1E/M3 cell culture and sample preparation

We established 4T1E/M3 mouse breast cancer cells from 4T1 cells (ATCC, Manassas, VA, USA) as described previously^31^,^32^,^33^. Cells were cultured in high glucose RPMI-1640 medium containing 10% fetal calf serum (FCS) and 20 mM HEPES at 37 °C under 5% CO_2_.

After adding culture medium (described above, 1.5 ml/dish) to the culture dish attached under the SiN-aluminium (Al)-holder, cells (4 × 10^4^; 20 μl/dish) were seeded and cultured at 37 °C under 5% CO_2_. The medium was changed after 2–3 days, and cells formed a sub-confluent or complete confluent monolayer on the SiN membrane in the holder after 4–5 days. Further, the Al holder with cells was separated from the plastic culture dish, attached upside down to another SiN film on an acrylic plate (15 × 15 mm) and sealed with an O-ring and four screws. The liquid sample holder containing the intact culture cells was finally attached to a sample stage with built-in pre-amplifier.

### Metal deposition on the upper SiN film

A 50-nm-thick SiN film supported by a 0.4 × 0.4-mm window in a Si frame (4 × 4 mm, 0.38-mm thick; Silson Ltd., UK) was coated with tungsten using a magnetron sputtering device (Model MSP-30T, Vacuum Device Inc., Japan). Tungsten was spattered for 15 s at 0.8-Pa argon pressure and 200 mA to produce a 15-nm-thick coating. The distance between the sputter target and SiN film was 50 mm.

### Dish sample holder and stage

Our dish sample holder comprised upper Al and lower acrylic resin portion that maintained the sample solution at atmospheric pressure between the SiN films (Fig. 1). The upper W-coated SiN film was attached to the Al holder using two-sided sticky tape (No. 7602, Teraoka Seisakusho Co., Ltd, Tokyo, Japan). The W layer on SiN film was connected to the Al holder using silver conductive ink (CW2900, ITW Chemtronics, Kennesaw, GA, USA). A hand-made Al holder (15 × 15 mm square) was attached under a 35-mm culture dish adhered with double-sided tape to a 4 × 4 mm square hole in the centre (Fig. 1a,a’). A 50-nm-thick SiN film in the 0.4 × 0.4 mm square window of a Si frame (4 × 4 mm) was fixed to the square hole in the culture dish bottom. The dish was subsequently UV sterilised for 17–18 h.

4T1E/M3 mouse breast cancer cells^31^,^32^ were cultured in the holder dish for 4–5 days as described above. Next, the Al holder containing cells and second SiN film on an acrylic plate were attached and sealed as described above (Fig. 1c,c’). The Al holder received voltage bias from four nickel–hydrogen batteries (approximately 8 V each), with a total bias voltage of approximately −32 V. The resin holder, which had high electrical resistivity, insulated the terminal underside of the holder from the metal-coated SiN film (Fig. 1d).

### High-resolution SE-ADM system and FE-SEM setup

The FE-SEM (JSM-7000F, JEOL, Tokyo, Japan) based high-resolution SE-ADM imaging system shown in Fig. 1d. The liquid-sample holder was mounted onto the SEM stage, and the detector terminal was connected to a voltage direct current (DC) pre-amplifier (1,000× gain) under the holder (Fig. 1c’). The temperature in the stage was approximately 28.2 °C, which was measured by a digital temperature indicator (CT-220, Custom Co., Tokyo, Japan). When the EB is used to irradiate the W-coated SiN, the negative potential increases in that area and is detected by the voltage DC amplifier through the measurement terminal. The electrical signal from the pre-amplifier was fed into the AD converter (AIO-163202FX-USB, CONTEC Co., Japan) after low-pass filtering (LPF; cut-off frequency 100 kHz). The LPF signal and EB scan signal were logged by a PC through an AD converter at a sampling frequency of 50 kHz SEM images (1,280 × 1,020 pixels) were captured at 2,000–10,000× magnification with a scanning time of 80 s, working distance of 7 mm, EB acceleration voltage of 7–10 kV and current of 10 pA.

### Optical phase microscopy and fluorescence imaging

Cultured 4T1E/M3 cells in 35 mm diameter glass bottom dish (Matsunami Glass Ind., Ltd., Osaka, Japan) were visualised at 400× magnification using an optical phase microscope (AXIO Observer A1; Carl Zeiss, Oberkochen, Germany). Fluorescent images of the ER and Golgi apparatus in culture cells were observed using a fluorescence filter of the excitation/emission wavelength of 480/534 nm after staining with the CytoPainter Golgi/ER staining kit (Abcam, Cambridge, MA, USA) according to the manufacturer’s protocol.

### Image processing

SE-ADM signal data from the AD converter were transferred to a personal computer (Intel Core i7, 2.8 GHz, Windows 7), and high-resolution SE-ADM images were processed from the LPF signal and scanning signal using Matlab R2007b software with an image processing toolbox (Math Works Inc., Natick, MA, USA). We initially observed a 3,840 × 1,020 pixel image using the AD converter of the SE-ADM system and a sampling frequency of 50 kHz. This initial image was converted to the correct size of 1,280 × 1,020 pixels or that of the corresponding SEM image. Corrected SE-ADM images were filtered using a two-dimensional (2D) Gaussian filter (GF) with a kernel size of 7 × 7 pixels and radius of 1.2σ. Background subtraction was achieved by subtracting SE-ADM images from the filtered images using a broad GF (400 × 400 pixels, 200σ).

### Monte Carlo simulation

Electron scattering in the W-coated SiN film was calculated via MC simulation using CASINO ver. 2.42^22^. The density and thickness of the W layer were 19.3 g/cm^3^ and 15 nm, respectively; the corresponding parameters of the SiN film were 3.12 g/cm^3^ and 50 nm, respectively. We estimated the density of culture medium was 1.012 g/cm^3^, because RPMI-1640 medium mainly included NaCl (6 g/L), NaHCO_3_ (1.5 g/L) and D-Glucose (4.5 g/L). The simulation parameters were set at 100,000 electrons, acceleration energy of 8 kV, and EB diameter of 3 nm. All MC simulations were performed on a personal computer (Intel Core i7 2.8 GHz, 8 G bytes RAM, Windows 7 operating system).

## Additional Information

**How to cite this article**: Okada, T. and Ogura, T. Nanoscale imaging of untreated mammalian cells in a medium with low radiation damage using scanning electron-assisted dielectric microscopy. *Sci. Rep*. **6**, 29169; doi: 10.1038/srep29169 (2016).

## Supplementary Material

Supplementary Information
