# Supplementary material for: Nanoscale imaging of untreated mammalian cells in a medium with low radiation damage using scanning electron-assisted dielectric microscopy
Source: Sci Rep. 2016 Jul 4;6:29169. doi: 10.1038/srep29169 (PMC4931576; doi:10.1038/srep29169)
Supplement: Supplementary Information [file srep29169-s1.pdf]

Supplementary information

Nanoscale imaging of untreated mammalian cells in a medium with low radiation damage using scanning electron-assisted dielectric microscopy

Tomoko Okada & Toshihiko Ogura*

Biomedical Research Institute, National Institute of Advanced Industrial Science and Technology (AIST), Central 2, Umezono, Tsukuba, Ibaraki 305-8568, Japan

*Corresponding author: Toshihiko Ogura

Biomedical Research Institute, National Institute of Advanced Industrial Science and Technology (AIST),

Umezono 1-1-1, Tsukuba, Ibaraki 305-8568, Japan

Tel.: +81-29-861-3408, Fax: +81-29-861-2677

E-mail: t-ogura@aist.go.jp

This PDF file includes:

Supplementary Figs 1 to 5

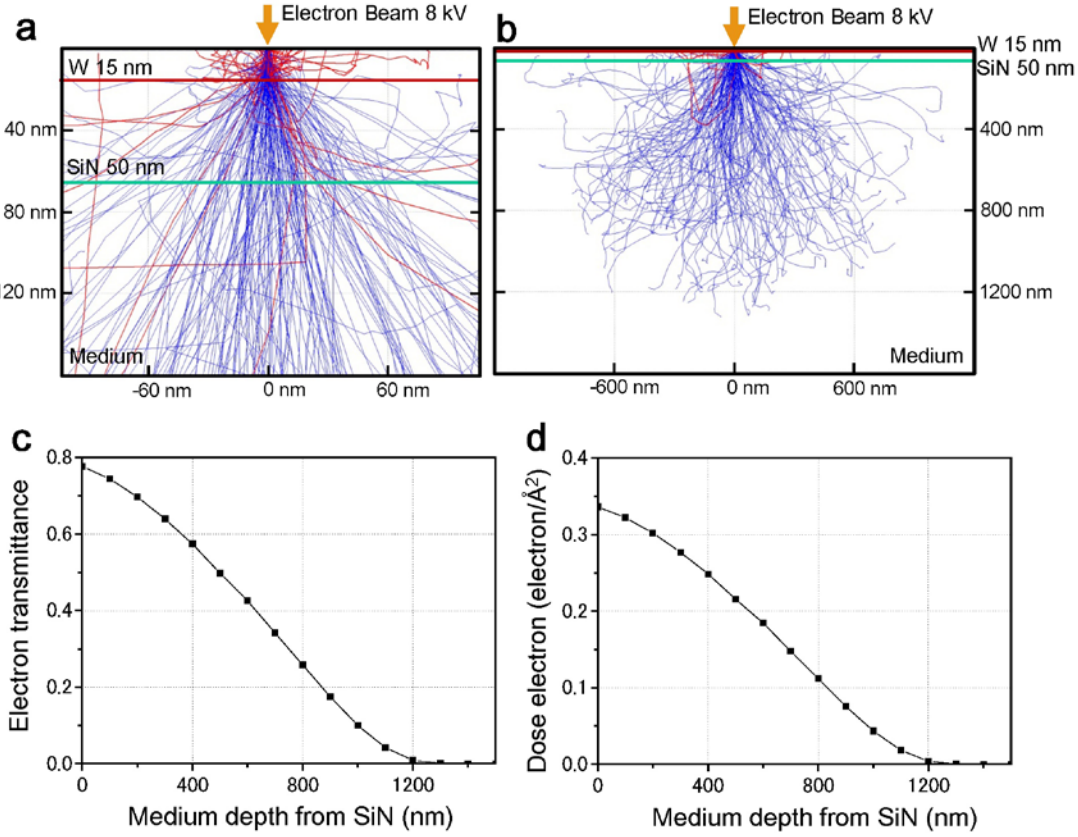

Supplementary Figure 1. Analysis of the electron trajectory in W-coated SiN film and medium in a Monte Carlo (MC) simulation. a, MC simulation analysis of W-coated SiN film, using CASINO ver. 2.42 (ref. 22); the electron trajectory area in a 15-nm W layer on 50-nm SiN film is shown. The respective densities of the W layer and SiN film were 19.3 g/cm^3 and 3.12 g/cm^3, and the respective thicknesses were 15 nm and 50 nm. The simulation parameters were set at 100,000 electrons, 8 kV accelerating voltage, and 3 nm EB spot diameter. **b**, Electron trajectories in W-coated SiN film with medium. Irradiated electrons were dispersed to a depth of almost 1 μm. **c**, Transmitted irradiated electrons in medium were calculated at each medium depth from the SiN film in a MC simulation. Electron transmittance just below the SiN film was 0.774; this value gradually decreased and reached 0 at a depth of 1.2 μm. **d**, The electron dose in medium was calculated at each EM condition and electron transmittance shown in (**c**), with a current of 10 pA, scanning area of 12 μm × 9.6 μm, and scanning time of 80 s. The electron dose just below the SiN film was 0.334 electron/Å^2; this dose gradually decreased and reached 0 electron/Å^2 at a depth of 1.2 μm.

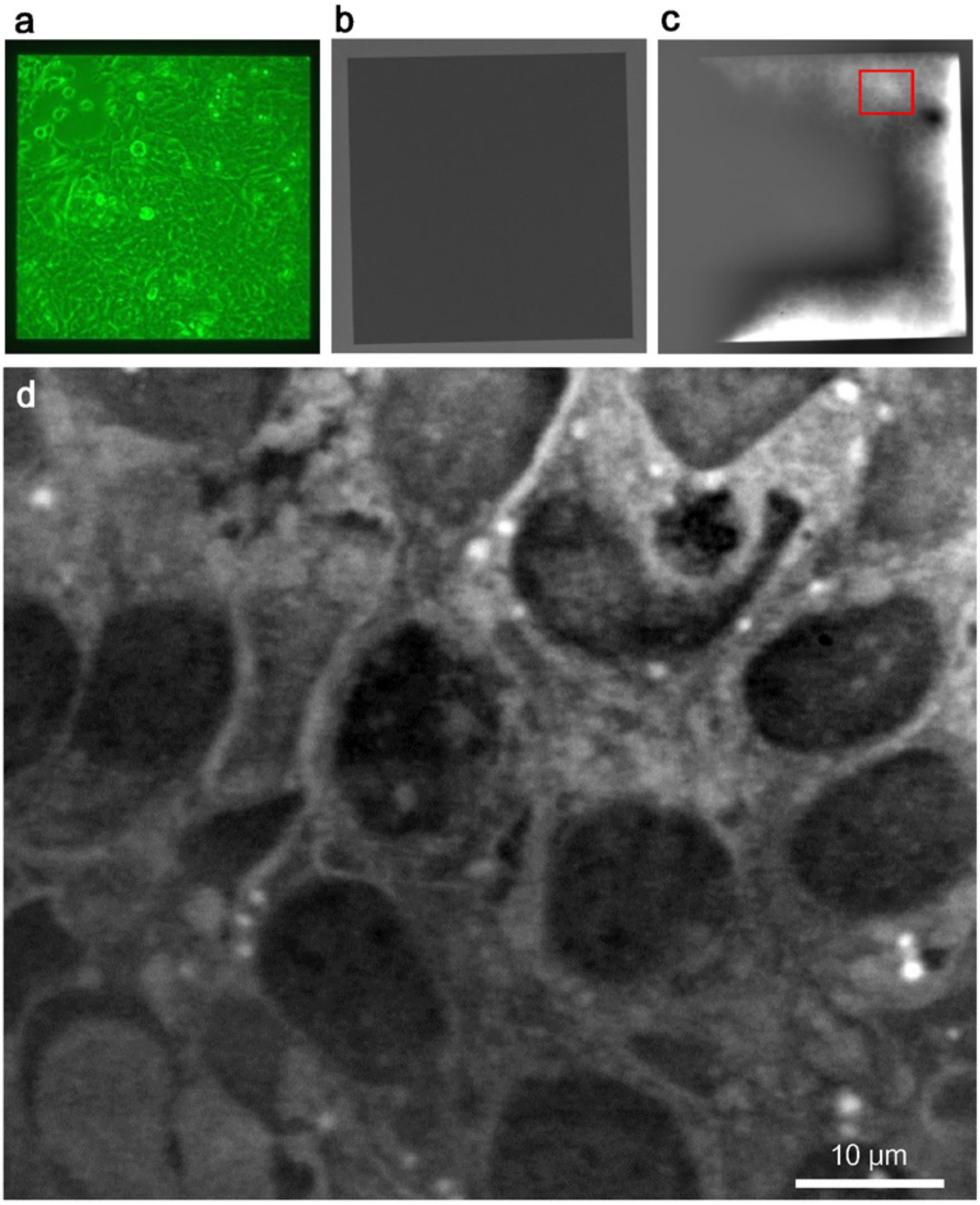

Supplementary Figure 2. Optical phase and dielectric observation images of the same cells. **a**, Optical microscopy image of cultured 4T1E/M3 cells in a confluent monolayer on SiN film in a dish holder. A SiN film size is 0.4 × 0.4 mm. **b**, Secondary electron detector-generated images of the same area as in (**a**). The image shows the SiN surface without cellular structures. **c**, Simultaneously observed dielectric image of the area in (**b**). The electric field signal was detected by the area of SiN film. **d**, High-magnification image of the red box in (**c**). Dielectric images clearly revealed cancer cells in the SiN square windows. The scale bar represents 10 µm in (**d**).

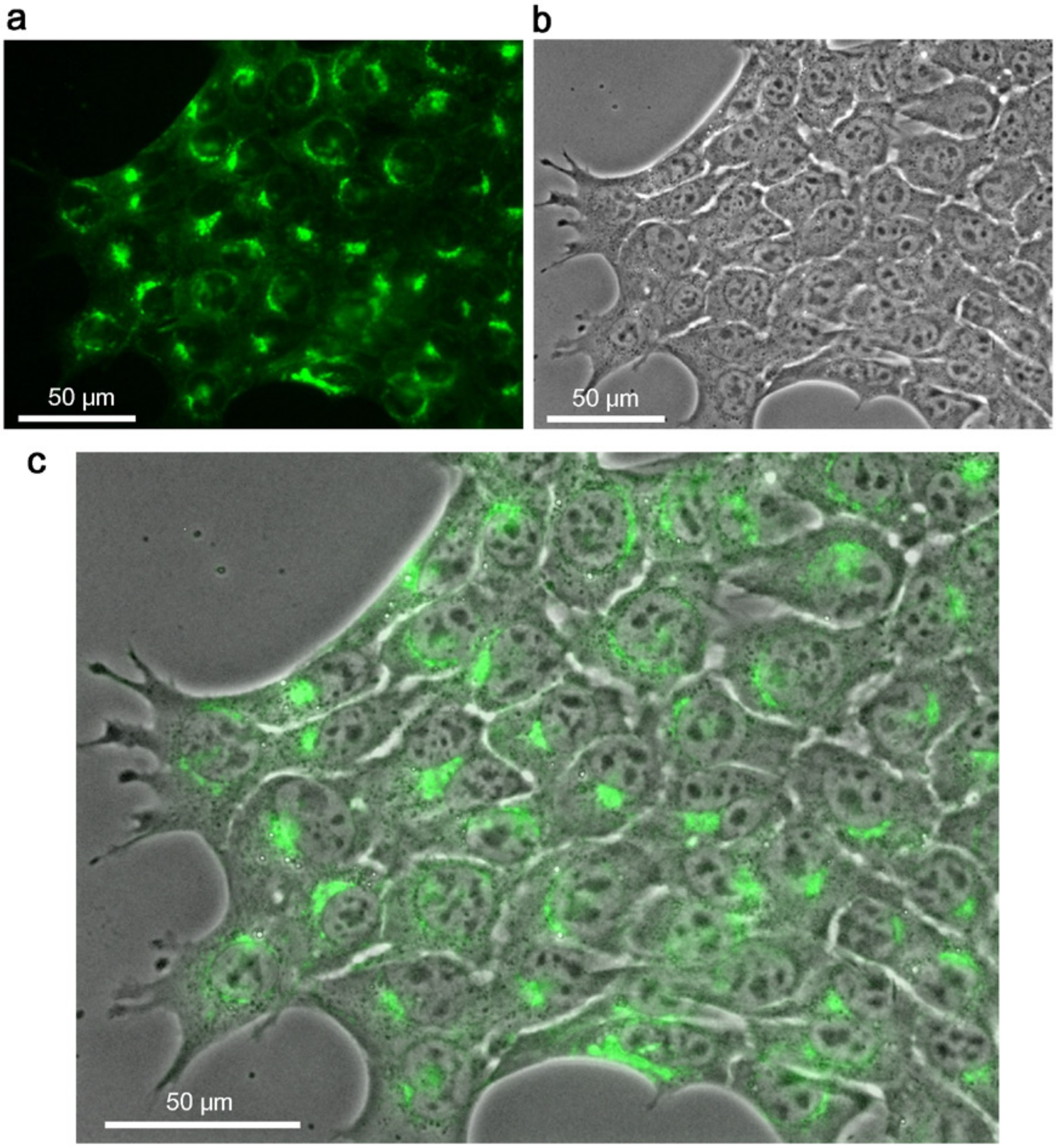

Supplementary Figure 3. Optical phase and fluorescence observation images of cells stained with Golgi/ER-specific dye. a, Fluorescence image of Golgi/ER-stained cells obtained from an optical microscope with a fluorescence filter. **b**, Optical phase contrast image of stained cultured cells obtained with an optical microscope. **c**, The merged picture of a Golgi/ER fluorescence image (**a**) and a phase contrast image (**b**). The ER and Golgi apparatus are localized near the cell nucleus. The scale bars represent 50 µm in (**a**) – (**c**).

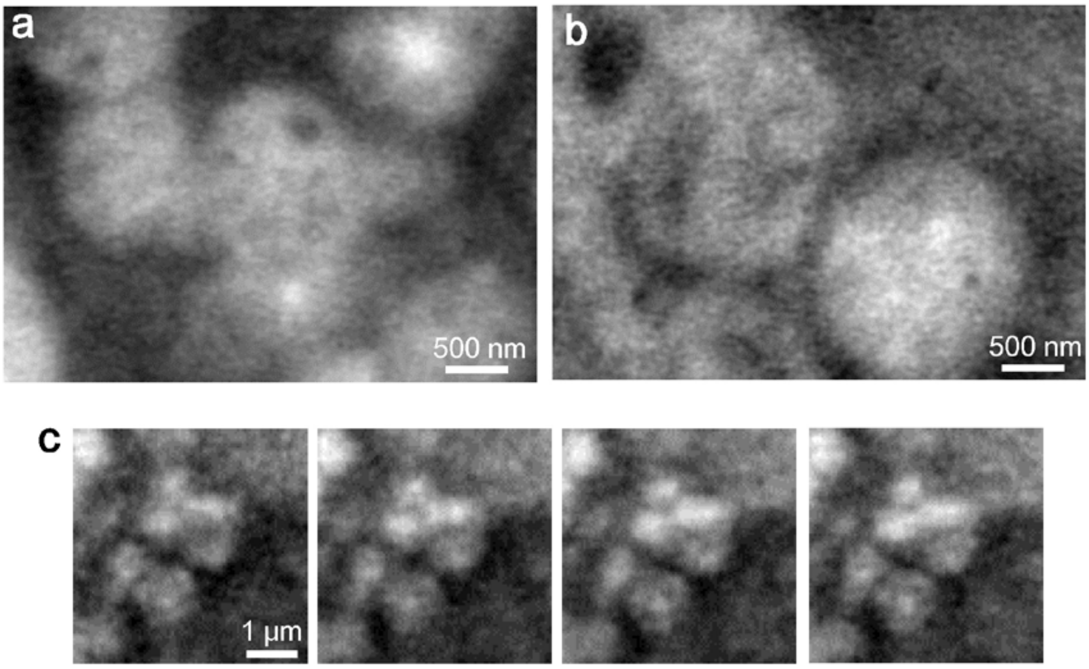

Supplementary Figure 4. Enlarged images of vesicle regions using SE-ADM. a, Enlarged images of the vesicle regions highlighted in red boxes in Figure 3b. **b**, Enlarged images of the vesicle regions highlighted in red boxes in Figure 3d**. c.** Enlarged images of a vesicle that exhibited contrast changes throughout the four observations indicated by blue arrows in Figure 4a–d. The scale bars represent 500 nm in (**a**) and (**b**) and 1 μm in (**c**).

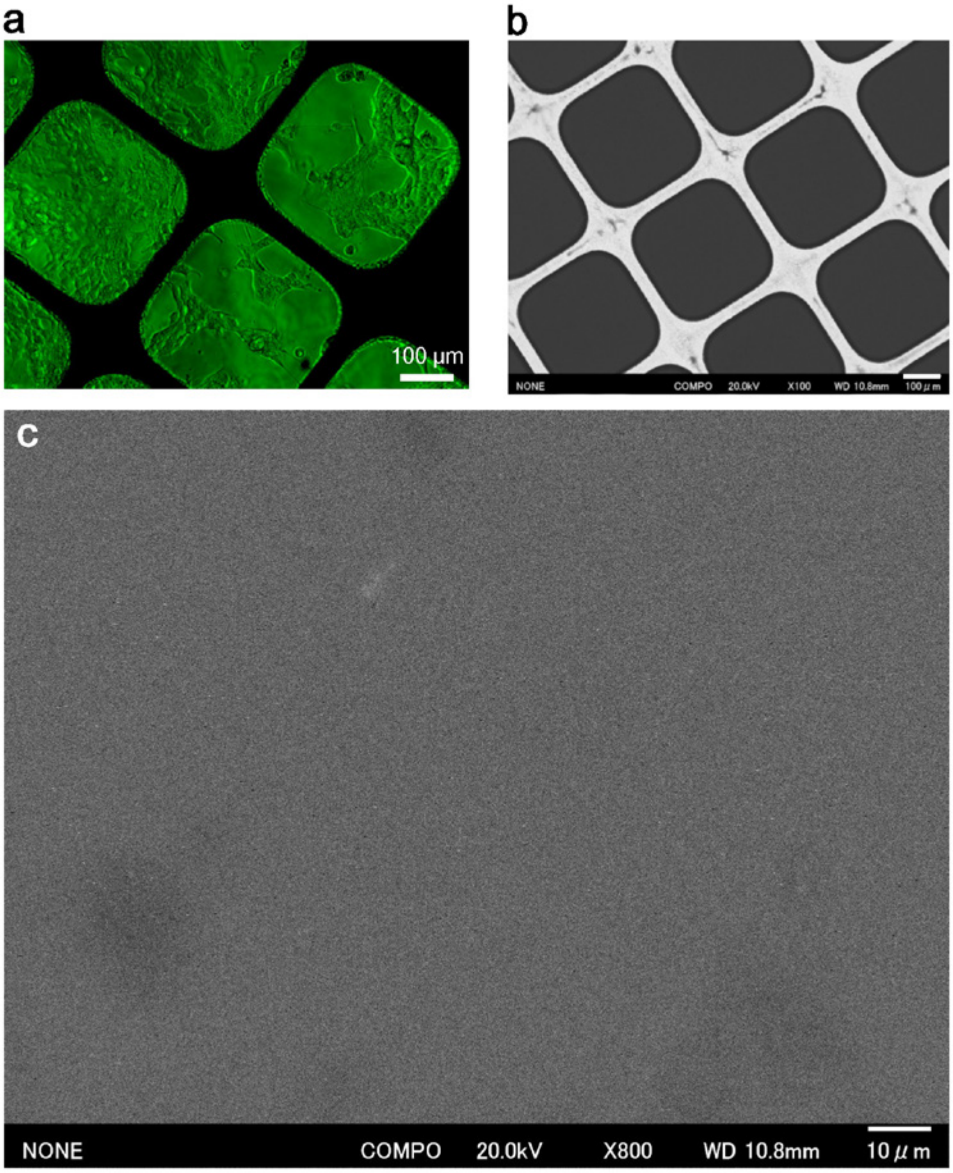

Supplementary Figure 5. FE-SEM observation of unfixed and unstained cells in a traditional atmospheric holder (Wet SEM). a, Phase-contrast optical image of a culture dish in the Wet SEM capsule (ref. 10). Untreated cells in medium are clearly visible in the wet capsule membrane by optical phase microscopy. **b**, Low-magnification FE-SEM image of the same cultured cells in the wet capsule shown in (**a**) with electron backscattering. The grid supporting the membrane is visible in this image. However, unstained cells cannot be detected. **c**, High-magnification image (800× magnification) with electron backscattering. The cell structures cannot be detected. The scale bars represent 100 µm in (**a**) and (**b**) and 10 µm in (**c**).
